# What Is behind In-Stream Advertising on YouTube? A Remote Neuromarketing Study employing Eye-Tracking and Facial Coding techniques

**DOI:** 10.3390/brainsci13101481

**Published:** 2023-10-19

**Authors:** Marco Mancini, Patrizia Cherubino, Ana Martinez, Alessia Vozzi, Stefano Menicocci, Silvia Ferrara, Andrea Giorgi, Pietro Aricò, Arianna Trettel, Fabio Babiloni

**Affiliations:** 1 Faculty of Economics, University of the International Studies of Rome, Via delle Sette Chiese 139, 00147 Rome, Italy; 2 BrainSigns Srl, Via Lungotevere Michelangelo 9, 00192 Rome, Italy; patrizia.cherubino@brainsigns.com (P.C.); ana.martinezlevy@uniroma1.it (A.M.); alessia.vozzi@uniroma1.it (A.V.); stefano.menicocci@brainsigns.com (S.M.); silvia.ferrara@brainsigns.com (S.F.); andrea.giorgi@uniroma1.it (A.G.); pietro.arico@uniroma1.it (P.A.); arianna.trettel@brainsigns.com (A.T.); fabio.babiloni@uniroma1.it (F.B.); 3 Department of Molecular Medicine, Sapienza University of Rome, Viale Regina Elena, 291, 00161 Rome, Italy; 4 Department of Anatomical, Histological, Forensic & Orthopedic Sciences, Sapienza University of Rome, 00185 Rome, Italy; 5 Unit of Histology and Medical Embryology, SAIMLAL Department, Sapienza University of Rome, 00185 Rome, Italy; 6 Department of Computer, Control, and Management Engineering, Sapienza University of Rome, 00185 Roma, Italy; 7 College of Computer Science and Technology, Hangzhou Dianzi University, Hangzhou 310005, China

**Keywords:** eye tracking, facial coding, visual attention, disgust, YouTube advertising, in-stream ads, neuromarketing

## Abstract

Not all elements displayed in a YouTube in-stream video ad are attributable to the ad itself. Some of those are automatically introduced by the platform, such as the countdown timer and the time progress bar. In recent years, some authors started exploring the effects associated with the presence of such non-ad items, providing valuable findings. However, objective evaluation of viewers’ visual attention is lacking in this context as well as emotional investigation. In addition, previous research showed how the manipulation of seemingly negligible details can yield dramatically different outcomes in the context of in-stream advertising. To extend knowledge, the authors explored the effects of the non-ad items’ presence by employing eye-tracking and facial coding techniques in combination with self-reports in a between-subjects experimental design focusing on the YouTube 15-s, mid-roll, non-skippable in-stream ad format. Results showed that the ad format currently employed by YouTube performs worse than its equivalent without the non-ad items on all the investigated measures and than its equivalent in which the non-ad items’ presence was experimentally reduced on facial coding disgust, self-reported disgust, ad irritation, and ad attitude. Managerial insights and challenges concerning the future of in-stream advertising and neuromarketing are highlighted.

## 1. Introduction

The growing worldwide availability of mobile devices, the accessibility of the Internet, and the introduction of video-based online platforms are the key factors that led to the high number of online video viewers worldwide we witness today, expected to reach nearly 3.5 billion by the end of 2023 [1,2]. Originally released to the public in 2005 to reduce the technical barriers encountered by non-expert users wishing to share videos on the web, YouTube has become one of the most influential social media platforms, ranking in second position among the most visited websites in the world, accounting for more than 2 billion monthly users and widely employed as a promotional tool by brands operating in different sectors [3,4,5].

Several studies have highlighted and discussed YouTube’s considerable marketing potential [6,7,8,9,10,11,12,13,14,15,16], among which it is worth mentioning a recent investigation of Wyzowl (2021) [17] who reported that 87% of video marketers employ YouTube for marketing purposes and found it to be effective, in contrast to other platforms such as TikTok that is used as a promotional tool by 20% of video marketers and considered effective only by 67% of them.

Similarly to Twitch and Facebook [18], YouTube services are provided at no cost to end users thanks to the company’s business model based on advertised-financed streaming services (AVoD), which strongly relies on ads available in multiple formats, such as skippable in-stream ads (also known as TrueView in-stream ads), non-skippable in-stream ads, in-feed video ads, bumper ads, outstream ads, and masthead ads [19,20]. In this context, skippable and non-skippable in-stream ads are the most popular formats, both playing in the video player, typically before the video starts (pre-roll ad) or by interrupting the video viewing experience (mid-roll ad) [21,22] and include several non-advertising elements, which are overlaid on the advertising content mainly to provide the viewer information about the ad duration and/or an option to skip the ad. In particular, whereas non-skippable video ads must be watched in their entirety before resuming the viewing experience [23,24], and can last 15 or 20 s depending on regional standards [25], the more recent skippable format, first released by YouTube in 2010, enables viewers to skip it after 5 s [26].

Although in-stream advertising represents an increasingly common choice for advertisers mainly due to its expected potential to attract the viewers’ attention [27], objective visual attention measurements in this context are lacking. In addition, the emotional investigation conducted in this context has mainly relied on self-reports so far, not exploiting the opportunities that lie behind additional techniques, such as those based on facial expression analysis (FEA), which have provided significant support in recent years to traditional measures in evaluating the emotional response to advertising [28,29,30,31]. Eye-tracker and facial coding, respectively employed for exploring consumers’ visual attention and emotion, are considered part of the neuromarketing research technologies [32,33,34,35], but in contrast to other methods traditionally used in neuromarketing, such as electroencephalography (EEG), functional magnetic resonance imaging (fMRI), functional near-infrared spectroscopy (fNIRS), and magnetoencephalography (MEG), which are referred to as brain-based techniques, they present several practical advantages. In particular, they can be used also to collect online data directly from the participants’ devices upon permission (desktop or smartphone) and without the need for the researcher to be physically close to research participants, implying a good level of confidence in generalizing experimental findings to the real world (ecological validity), as well as several additional benefits related to the management of data collection (e.g., quick non-moderated tests and worldwide coverage). In addition, the recent COVID-19 pandemic had a substantial impact on the widespread adoption and advancement of remotely administered techniques for data collection, including webcam-based eye tracking and facial coding, whose standards of accuracy are increasingly approaching those associated with their traditional and “in-lab” counterparts [36,37,38].

By employing remote webcam-based eye-tracking and facial coding, this study has the primary objective of verifying whether the presence of non-advertising items overlaid on the ad content (e.g., the timer or countdown) affects visual attention and time to notice associated with key ad elements (e.g., brand and product), and moral disgust detected by facial coding during the view of the YouTube 15-s, mid-roll, non-skippable in-stream ad format. Moreover, to get a more complete picture, similar effects are investigated on traditional self-reported measures typically employed in advertising research, such as ad irritation, ad avoidance, and ad attitude, as well as on self-reported disgust. The following sections will present the main findings related to the framework of interest, current limitations, the rationale underlying the study and specific choices (e.g., investigation restricted to a specific in-stream ad format), and main research questions.

### 1.1. Visual Attention and In-Stream Video Ads

Although visual attention is known to serve as a precursor to the advertising impact [39], as consumers need to pay attention to ads in order to process related information [40,41], online ads often tend to be visually overlooked [42].

Ad avoidance, in the digital context, is defined as “any action that reduces exposure, or the turning off, to digital advertising” [43], and represents a severe issue for advertisers due to its potential to drastically decrease the effectiveness of their investments [44,45,46,47,48]. To provide an indication of the magnitude of this phenomenon, it is worth pointing out that more than 50 percent of online ads sponsored by companies are never viewed by end users [49]. Among the avoiding strategies typically employed by online users, it is worth mentioning the extensively studied “banner blindness”, a term coined by Benway in 1998 [50] to indicate the tendency to avoid display advertising due to the “increasing abilities of users in identifying banner ads and their persuasive purposes” [51]. Fortunately for advertisers, new advertising strategies have been developed over time to address similar issues, as evidenced for instance by the introduction of native ads, more difficult to recognize by users due to the similar look and feel of the media format in which they are integrated [52,53,54,55,56,57].

Although in-stream video ads have proven to increase the focus on the advertising content in comparison to other ad formats [58], viewers dealing with in-stream video ads can adopt simple strategies to avoid advertising content and its persuasive purposes. In particular, in-stream video ads viewers’ visual attention may shift out of the screen or toward non-ad-related content, such as the video player’s controls, the timer, the skip button, and if the player is not in full-screen mode, also towards recommendations for similar content, titles, and comments [59]. However, the users’ capacity to adapt to, identify, and ignore digital advertising [57,60,61,62,63,64,65,66] followed by avoiding behaviors, such as shifting attention towards regions of the screen where the advertising content is not salient [67,68], is not the only factor leading to reduced visual attention towards the advertising content. In this direction, an eye-tracking study conducted by Kim et al. (2023) [68] on mobile in-stream video ads suggested that, while watching non-skippable ads, viewers’ visual attention tends to highly focus on the timer because viewers aim to figure out how long they are “forced” to watch the ad before resuming the original video, reflecting the need for temporal certainty. Moreover, dynamic or animated items, as is the case of the timer in in-stream video ads, have been shown to attract higher attention than static ones according to several studies focused on digital advertising [65,69,70,71]. Remarkably, the focus on similar secondary tasks is likely to limit the processing of relevant advertising information (e.g., brand information) and decrease the advertising efficacy [41]. In addition, as emphasized by Zhang et al. (2018) [72] in their study concerning video advertising, visual attention toward key advertising elements such as brand, product, and endorser plays a key role, as it positively affects attitude toward the ad, attitude toward brand, and purchase intention. In the context of in-stream video ads, where the objective evaluation of visual attention is lacking, it is crucial to highlight the study by Frade et al. (2022) [59], which compared the impact of skippable and non-skippable in-stream ads. The authors took advantage of eye-tracking technology in a lab experiment based on a between-subjects experimental design involving 157 subjects who were invited to take part in a task consisting of a viewing experience on a website that resembled YouTube. The results provided by Frade and colleagues revealed that viewers exposed to skippable in-stream ads tend to focus their attention on the location of the skip button for an average of 40.59% of the ad duration in contrast to 12.43% associated with participants exposed to non-skippable in-stream ads. Furthermore, the authors reported a more positive ad attitude, higher brand recall, and lower intrusiveness when subjects were exposed to non-skippable in-stream ads in comparison to skippable in-stream ads, leading to the overall conclusion that non-skippable in-stream ads are significantly more effective than skippable in-stream ads. Among the valuable findings reported by Frade and colleagues, it is worth noting a further insight: regardless of the specific format, mid-roll in-stream ads, responsible for interrupting the viewing video experience, were perceived as more intrusive and generated a more negative ad attitude in comparison to pre-roll in-stream ads.

Taking into account the aforementioned findings, which suggested the relevance of investigating the visual attention elicited by key advertising elements (e.g., brand and product) [72], the superiority of the non-skippable format in enhancing visual attention towards ad content in general, and the critical issues associated with the position mid-roll [59], the following research question arises:

- RQ1: Does the presence of elements not related to advertising and overlaid on the advertising content (non-ad items’ presence) affect visual attention and time to notice associated with key ad elements (e.g., brand and product) during the view of the YouTube 15-s, mid-roll, non-skippable in-stream ad format?

For the sake of clarity, it is crucial to note that the 15-s duration associated with the non-skippable ad format is the regional standard for Italy, the country where the study has been conducted [25,73].

### 1.2. Emotions, Disgust, and In-Stream Video Ads

It has been four decades since Zajonc revealed the significant function of emotion in advertising [51,74], leading subsequent research to seriously take into account the opportunities underlying the potential of advertising in targeting senses and emotions [75,76,77]. Many studies have suggested that emotions play a crucial role in determining communication effectiveness [78,79,80,81] and, concerning social media and online video advertisements, emotions have been often identified as a precursor to consumer attitudes toward the ads [82,83,84]. Several authors pointed out disgust, a regulatory human emotion linked to particular physiological reactions and a distinctive facial expression [85,86,87], as a crucial emotional mediator of advertising effects [88,89,90,91].

The food and beverage sector, where firms allocate significant financial resources each year towards the promotion of their goods, amounting to billions of dollars [92], is traditionally acknowledged as a key context to investigate the impacts of emotions of disgust, which has been often referred as a food-related emotion [93] and whose negative effects on consumer behavior have been widely documented [94,95,96]. In addition, it is worth noting that with the recent rise in popularity of insect food products in the Western world, disgust-based research experienced significant growth [97,98,99,100,101].

However, limiting the scope of disgust to its association with food, as a mere food-related emotion, is highly inaccurate, as the fundamental role of moral disgust would be ignored. In contrast to core disgust, which occurs in response to physical or chemical stimuli, moral disgust arises in response to abstract violations of moral norms which can be further divided into bodily violations (e.g., sexual taboos) and nonbodily violations (e.g., betrayal or deception) [102,103]. While moral disgust associated with bodily violations has received considerable attention in advertising, in particular by studies focused on controversial advertising [104] which demonstrated its negative effect on ad attitude [105] and even behavior [106], the role of moral disgust associated with nonbodily violations has been far less taken into account in advertising research. In this context, it is crucial to make a further clarification: whereas moral disgust occurring in response to nonbodily violations (e.g., betrayal or deception) is also often associated with anger, manifestations of disgust in response to bodily violations (e.g., sexual taboos) are typically not [103]. The use of facial coding in scenarios where disgust may be in part contaminated by anger (nonbodily violations) is particularly suitable because facial expressions of moral disgust are sensitive also to moralistic anger [107], and in general, disgust tends to exhibit higher levels of accuracy in facial coding detection when compared to other emotions such as anger [108]. Another issue that deserves attention concerns irritation, which more positively correlates to disgust than anger state [109] and positively affects ad avoidance, as shown by a study conducted on YouTube in-stream video ads [110]. These patterns of relationships seem to find further support in the origin of disgust itself, as disgust has been suggested to have originated as a defense mechanism aimed at safeguarding the organism against potential external threats and potentially leading to avoidance tendencies [111,112,113,114,115].

In light of these factors and their implications in in-stream advertising, it is crucial to consider that in-stream video ad viewers, being forced to watch the ads for a given duration (skippable format) or in their entirety (non-skippable format), may feel “deceived” (nonbodily violation), as well-summarized by a study participant dealing with in-stream ads: “You can’t force me to watch those ads!” [116]. Moreover, in line with advertising theories on intrusiveness and with several studies investigating the forced exposure to online ads [117,118,119,120,121,122], individuals experience irritation towards advertisements that show up unexpectedly and interfere with the original goal (e.g., viewing the selected video). Since similar violations of moral norms are capable of eliciting moral disgust, irritation, and/or avoidant tendencies according to the above-mentioned framework, it would be valuable to investigate whether the presence of non-ad items overlaid on the ad content contributes to reducing or increasing the magnitude of such undesirable potential outcomes. In this direction, a study conducted by Jeon et al. (2019) [123] on pre-roll non-skippable in-stream video ads suggested that the presence of the timer, a key non-ad-element overlaid on the ad content that reflects cognitive control and temporal certainty, increases ad irritation in the case of 30-s or 60-s ads while reducing it when the ads last 15-s. Based on these findings, it seems clear how temporal certainty may affect ad irritation in different directions, depending on specific features of the in-stream ad (e.g., ad length in the context of pre-roll in-stream ads). The relevance of taking into account specific features in the investigation of in-stream ads has been also emphasized by Frade et al. (2022) [59], who revealed that ad position can significantly affect key results concerning in-stream video ads provided through YouTube. In particular, the authors reported that mid-roll ads were characterized by higher scores on ad intrusiveness and elicited a more negative ad attitude than pre-roll ads. Furthermore, due to the high popularity of YouTube, which is the second most visited website in the world [4], it is reasonable to assume that users are becoming increasingly familiar with YouTube in-stream ad formats, whose advertising intent is made extremely clear, salient, and recognizable by the presence of the non-ad items overlaid on the ad content. Accordingly, one may argue that the presence of the non-ad-elements, clearly revealing the advertising intent through a familiar design, would reflect a transparent act of loyalty toward the viewer capable of mitigating the viewer’s feeling of having been deceived (unexpected interruption of the selected video and forced ad fruition) and leading therefore to reduced moral disgust, ad irritation, ad avoidance, and/or more favorable ad attitude.

However, the theories underlying memory, emotion, and neural interconnections in semantic networks may suggest a radically different interpretation in this context, since the activation of an object’s representation is known to trigger associated and automatic emotional responses, attitudes, attributes, and concepts independently of the reasoning [124,125,126,127]. Similarly, as the non-ad items overlaid on the ad content represent the most distinctive feature of an ad format that disrupts the viewing experience and forces the exposure to the ad, their presence, at least in the familiar design employed by YouTube, is likely to symbolize not only the most salient cue of a deceptive practice but deception itself, potentially leading to unfavorable outcomes on most of the measures of interest.

Based on this framework and narrowing down the focus of the investigation to the YouTube 15-s, mid-roll, non-skippable in-stream ad format, given the popularity of the platform [4], the duration standards [25,73], the opportunity to extend previous valuable findings currently limited to pre-roll ads [123], and the relevance of the non-skippable format [59] where the moral violation may be highly salient due to the prolonged forced exposure [116], we aimed to investigate the effect related to the presence of the non-ad items on several measures of interest, by addressing the following research questions:

- RQ2: Does the presence of elements not related to advertising and overlaid on the advertising content (non-ad items’ presence) affect the occurrence of facial expressions of moral disgust detected during the view of the YouTube 15-s, mid-roll, non-skippable in-stream ad format?
- RQ3: Does the presence of elements not related to advertising and overlaid on the advertising content (non-ad items’ presence) affect the self-reported measures of ad irritation, ad avoidance, ad attitude, and moral disgust concerning the view of the YouTube 15-s, mid-roll, non-skippable in-stream ad format?

## 2. Materials and Methods

### 2.1. Participants

The study involved 175 participants (91 females and 84 males; mean age = 25.8 years; SD = 5.7 years) recruited on a voluntary basis, who were properly informed and instructed about the remote study and signed a digital informed consent. Data were managed following standard practices and in compliance with the GDPR and the European Code of Ethics for Research, and the university’s ethical committee approved it. The experiment was also performed in accordance with the principles outlined in the Declaration of Helsinki of 1975, as revised in 2013. Participants did not receive compensation for their research participation.

### 2.2. Experimental Conditions and Stimuli

The study was based on a between-subjects experimental design, which involved assigning participants to three different conditions defined as “Absent”, “Current”, and “Low”, according to the presence of the non-ad items overlaid on the ad content of the in-stream ad they were exposed to, that, in line with our objectives, was a YouTube 15-s, mid-roll, non-skippable in-stream ad format.

From this moment on, for the sake of brevity, the “YouTube 15-s, mid-roll, non-skippable in-stream ad format” could be simply referred to as the ad. In order to preserve a similar sample size across conditions, a computer-based randomization procedure based on the random assignment by blocks method was used (“Research Randomizer” tool, available at the following link: https://www.randomizer.org (accessed on 6 January 2023)). As each subject could participate remotely in the study through either a desktop or smartphone device, the same computer-based randomization procedure was employed to ensure a similar sample size between desktop and smartphone users within each condition. Accordingly, the sample was distributed across the three conditions as follows: “Absent” (Total: n = 57; Desktop: n = 28; Smartphone: n = 29), “Current” (Total: n = 62; Desktop: n = 32; Smartphone: n = 30), “Low” (Total: n = 56; Desktop: n = 28; Smartphone: n = 28). More details about the technical difficulties that prevented a perfect balance in terms of sample size across conditions or within conditions as well as deeper analysis at the device level are provided in the discussion section.

In contrast to the “Current” condition, where the “non-ad items’ presence” resembled exactly that of the 15-s, non-skippable in-stream ad format currently employed by YouTube, viewers assigned to the “Absent” condition viewed the ad without non-ad items in overlay to the ad. The viewers assigned to the “Low” condition were instead exposed to an alternative non-ad items design, where the presence of the latter was reduced and customized in comparison to the “Current” condition. Meticulous work of video editing and motion graphics was performed to ensure that the ad format serving the “Current” condition perfectly matched the 15-s non-skippable ad format currently used by YouTube, in all its elements. To successfully accomplish this task, two references, respectively for desktop and smartphone views, were identified on YouTube and video recorded. After a detailed inspection of the non-ad items design associated with each reference, an identical non-ad items design was created, paying crucial attention to both style and behavior (e.g., animation). This step was crucial, as from this point on we would be able to overlay YouTube’s non-ad items onto the advertising content of interest. As the ad content selected for this study was already available in the smartphone YouTube reference but could not easily separated by the overlaid non-ad-items, we found it easier to download its version, free of any non-ad items, directly from the YouTube channel of the Italian company that designed the ad [128]. Having obtained at this stage an isolated version of YouTube’s non-ad items for both desktop and smartphone views, as well as the isolated ad content of interest, we were able to develop the ad stimuli serving the “Current” condition for both desktop and smartphone views by overlying the YouTube’s non-ad items on the ad content. Once the ad stimuli for the “Current” condition were defined, we developed the stimuli serving the “Absent” condition, which included only the ad content free of any non-ad items. Finally, we designed the ad stimuli for the “Low” condition, where the presence of the non-ad items was reduced and customized. At the end of this process, to replicate the ordinary YouTube viewing experience, the developed ad stimuli were integrated into a selected video, in the mid-roll position. In contrast to the non-ad items’ presence which varied across conditions, the selected ad content and the video did not. The overall process of video editing and motion graphics was performed by using Adobe Premiere Pro CC 2019 and Adobe After Effects CC 2019 (version 16.1, Adobe Systems Software Ireland Limited, Dublin, Ireland).

The video employed in this study was a real YouTube tutorial video explaining how to make a microwave mug cake, while the ad content was, as already mentioned, a real non-skippable ad found on YouTube and aimed at sponsoring the Ringo doughnut-shaped biscuits (a well-known product in Italy).

To inspect in detail the non-ad items’ presence serving each condition of interest, compare the original references to the final ad stimuli (quality check), and/or view an example of the entire YouTube viewing experience including the video and the mid-roll ad, please see Appendix A.

### 2.3. Experimental Protocol

**Figure 1:**
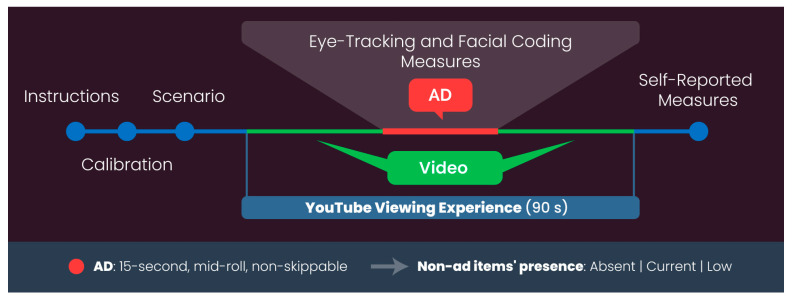
Experimental protocol.

The key phases of the experimental protocol adopted for this study are illustrated in Figure 1.

At the beginning of each experimental session, participants received some technical instructions aimed at improving the accuracy of eye-tracking and facial coding data collection such as “Please make sure you do have not any strong light source behind you” and “Please make sure your face is well illuminated”.

After a short calibration process, necessary to enable eye-tracking and facial coding data collection, participants were given the following scenario aimed at replicating an online learning experience on YouTube [129,130,131,132]: “You are interested in learning how to make a mug cake in the microwave, so after visiting YouTube and selecting a short video tutorial that explains how to make it, you are now ready to watch the video (click this button to watch the video)”. After the instruction, participants began taking part in the YouTube viewing experience in full-screen mode. The overall YouTube viewing experience lasted 1 min and 30 s where the first 46 s were dedicated to the video, the following 15 s to the mid-roll non-skippable ad, and the final 29 s to the video again. In this context, it is worth remembering that depending on the specific condition the participant was exposed to (“Absent”, “Current” or “Low”), the presence of the non-ad items varied.

Once the YouTube viewing experience was concluded, the self-reported measures of interest were collected (disgust, ad irritation, ad avoidance, ad attitude), as well as other self-reported measures needed to control confounding in the analysis (YouTube video viewing frequency, food ads attitude, product attitude, product purchase frequency). For more details about self-reported measures, see Appendix A.

### 2.4. Data Collection and Performed Analysis

Data collection was performed remotely through both Qualtrics (Qualtrics, UT, USA) and Sticky software (2023 version, Tobii, Stockholm, Sweden). In particular, by integrating a Sticky script in the Qualtrics environment, it was possible to enable eye-tracking and facial coding data recording using the participants’ webcam [133].

Whereas Qualtrics was mainly used for the collection of self-reported measures of interest, the viewers’ visual behavior and emotional reaction were recorded and processed through the Sticky automated cloud-based platform, an online solution that allows researchers to perform online and webcam-based eye tracking and facial coding [134,135,136,137]. This software does not require humans to manually code facial expressions; instead, it allows automatic facial expression detection and classification taking advantage of Ekman’s facial action coding system (FACS) [138] and Picard’s algorithms [108,139], similar to other well-known automatic facial coding software such as Noldus’s Face Reader (version 9.1, Noldus Information Technology bv, Wageningen, The Netherlands) and Affectiva (Affectiva, Waltham, MA, United States).

The emotional investigation was conducted on raw facial coding data, which returned the occurrence of facial expressions relating to disgust out of the total facial expressions detected and therefore expressed in percentage terms, in reference to each frame analyzed (4 frames per second). The investigation was limited to the time window of the advertisement which lasted 15 s, and for each subject, the percentage of disgust detected was averaged for all the frames analyzed. As a result, an average value was obtained for each subject, indicating the average percentage of disgust-related facial expressions detected while viewing the ad (RQ2).

Regarding the eye-tracking investigation, sessions providing low-quality data due to factors such as bad light conditions, too dark or too strong background light, and excessive and large head movements were automatically excluded by Sticky’s algorithm [140]. In addition, the calibration process, requiring participants to follow a circle moving across multiple regions of the screen, is another crucial factor employed by the system for the quality check. In particular, calibration quality is validated by taking into account how much the gaze prediction is different from the ground truth value for the validation points and whether the difference is higher than a threshold value of 0.18, the session is automatically excluded [140].

The eye-tracking investigation was performed at 15 Hz and was based on the visual behavior of viewers detected during the advertising, taking into consideration several areas of interest (see Appendix A for a detailed overview of the AOIs). In this direction, and according to RQ1, the primary focus of the eye-tracking investigation was placed on the AOIs related to the key ad elements (brand and product items). Two eye-tracking metrics, referred to as total visual attention (%) and viewable to seen (s), were employed to assess viewers’ gaze behavior. The total visual attention, also called “total viewing time” [141] or “total visit duration” [142], reports the total amount of time the participant spent looking at an AOI, and like other similar eye-tracking metrics, once divided by the time frame of interest (total ad duration in our context) and expressed in percentage, provides the advantage of maintaining a strong connection with the study context [18,51]. To address RQ1, the total visual attention associated with the “key ad elements” was computed for each participant as the sum of the total visual attention scores obtained for each “key ad element” of interest. The “viewable to seen” metric reports the average amount of time in seconds from the moment an AOI is viewable on the screen until the participant sees it [143]. After having obtained the “viewable to seen” scores for each key ad element, the scores related to the “key ad elements” AOI group were computed by averaging the scores of their children AOIs. Such a process provided, therefore, the average time in seconds a participant spent viewing the key ad elements. Despite our primary objectives relying on the key ad elements, to provide additional cues about the viewers’ gaze behavior, we additionally computed the total visual attention (%) and viewable to seen (s) metrics for the non-ad items, following the same procedure previously described for the key ad elements.

The self-reported measures of interest were selected according to the advertising literature relevant to the current framework. In particular, along with the measures of ad irritation [123,144], ad avoidance [23,110], and ad attitude [145,146] that are traditionally and widely employed in advertising research, self-reported disgust [104,105], although less popular than the previous ones, was taken into consideration as a further useful indicator to describe the entity of the violation [102,103,104,105,106]. In order to mitigate biases potentially arising from the modalities underlying self-report data collection performed through Qualtrics, counterbalancing was employed.

The analysis aimed to explore the effect of the non-ad items’ presence on the visual attention and time to notice associated with key ad elements (RQ1), facial coding disgust (RQ2), and other several self-reported measures of interest, such as disgust, ad irritation, ad avoidance, and ad attitude (RQ3) while controlling for the potential confounding effects induced by additional variables (YouTube video viewing frequency, food ads attitude, product attitude, product purchase frequency). As the variables of interest were not non-normally distributed and included ordinal variables (e.g., self-reported measures), non-parametric tests were employed [147]. In particular, the non-parametric ANCOVA, also known as Quade’s test [148], was employed to address all our research questions, after having verified that the covariates (YouTube video viewing frequency, food ads attitude, product attitude, product purchase frequency) had the same distribution across all levels of the factor (non-ad items’ presence), which is a crucial assumption of this statistical test [148,149]. Moreover, for the following post hoc pairwise comparisons, Scheffe’s adjustments were applied, as the sample size across conditions did not perfectly match (“Absent”: N = 57; “Current”: N = 62; “Low”: N = 56) [150]. The overall analysis process was performed in SPSS (IBM SPSS Statistics 22, IBM, Armonk, NY, USA), and JASP (version 0.17.3.0, JASP Team, Amsterdam, The Netherlands).

## 3. Results

### 3.1. Total Visual Attention and Viewable to Seen—RQ1

**Figure 2:**
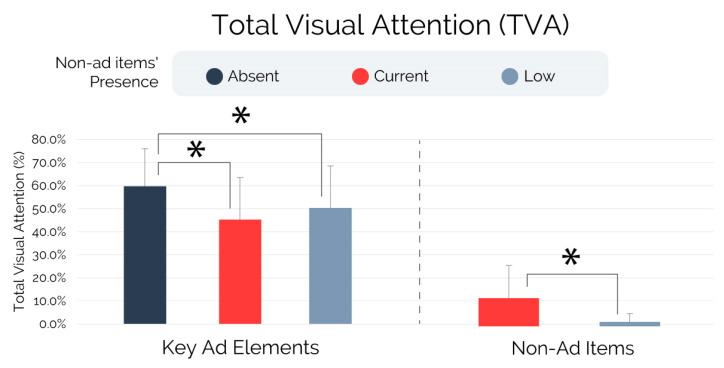
Total visual attention—key ad elements and non-ad items. Note: Error bars represent standard deviation. Asterisks indicate statistically significant differences for *p* < 0.005.

The analyses performed on the total visual attention, illustrated in Figure 2 and based on the non-parametric ANCOVA, showed a significant effect of non-ad items’ presence on both the total visual attention elicited by the key ad elements (F = 8.757, *p* < 0.001, r^2^ = 0.099) and non-ad items (F = 48.393, *p* < 0.001, r^2^ = 0.309). Whereas the results obtained for the non-ad items were limited to the “Current” and “Low” conditions (non-ad items were not available in the “Absent” condition) and indicated that subjects exposed to the “Current” condition paid a significantly higher overall amount of visual attention to the non-ad items in comparison to the “Low” condition, multiple pairwise comparison tests (post hocs) were additionally performed concerning total visual attention elicited by the key ad elements. In this direction, the analysis revealed that subjects exposed to the “Absent” condition exhibited significantly increased total visual attention towards the key ad elements than subjects exposed to the “Current” (p_scheffe_ < 0.001, r = 0.786) and “Low” conditions (p_scheffe_ = 0.022, r = 0.542). No statistical differences were detected while comparing the total visual attention elicited by the key ad elements between the Current” and “Low” conditions (p_scheffe_ = 0.442).

**Figure 3:**
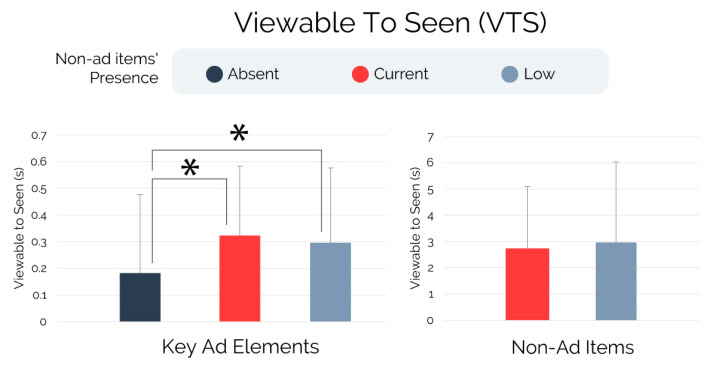
Viewable to seen—key ad elements and non-ad items. Note: Error bars represent standard deviation. Asterisks indicate statistically significant differences for *p* < 0.005.

The analyses performed on the “viewable to seen” metric, represented in Figure 3 and based on the non-parametric ANCOVA, showed a significant effect of non-ad items’ presence on the viewable to seen scores associated with the key ad elements (F = 6.291, *p* = 0.002, r^2^ = 0.074), while no effect was found on the viewable to seen scores associated with the non-ad items (F = 0.349, *p* = 0.556). Concerning the viewable to seen scores associated with the key ad elements, post hoc tests revealed that subjects exposed to the “Absent” condition exhibited significantly decreased viewable to seen scores than subjects exposed to the “Current” (p_scheffe_ = 0.004, r = −0.655) and “Low” conditions (p_scheffe_ = 0.038, r = −0.502). No statistical differences were detected while comparing the “Current” and “Low” conditions on the viewable to seen scores associated with the key ad elements (p_scheffe_ = 0.732).

### 3.2. Facial Coding (FC) Disgust—RQ2

**Figure 4:**
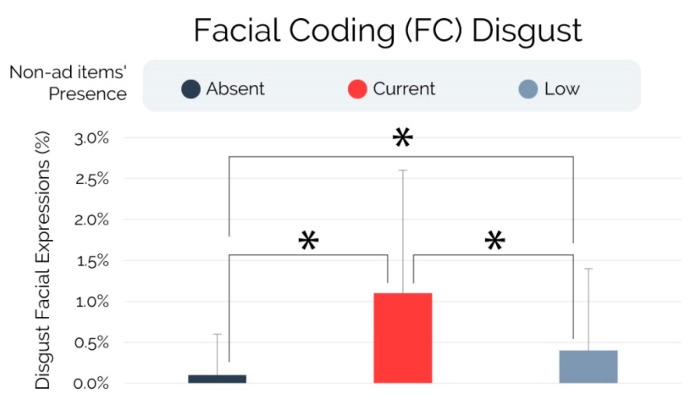
Facial coding (FC) disgust. Note: Error bars represent standard deviation. Asterisks indicate statistically significant differences for *p* < 0.005.

The analyses performed on the facial expressions of disgust, illustrated in Figure 4 and based on the non-parametric ANCOVA, showed a significant effect of non-ad items’ presence on the facial coding (FC) disgust (F = 16.711, *p* < 0.001, r^2^ = 0.174). Multiple pairwise comparison tests (post hocs) revealed that subjects exposed to the “Absent” condition exhibited a significantly lower occurrence of facial expressions of disgust than subjects exposed to the “Current” (p_scheffe_ < 0.001, r = −1.113) and “Low” conditions (p_scheffe_ = 0.022, r = −0.542). In addition, subjects exposed to the “Low” condition exhibited a significantly lower occurrence of facial expressions of disgust than subjects exposed to the “Current” (p_scheffe_ = 0.013, r = −0.571).

### 3.3. Self-Reports—RQ3

**Figure 5:**
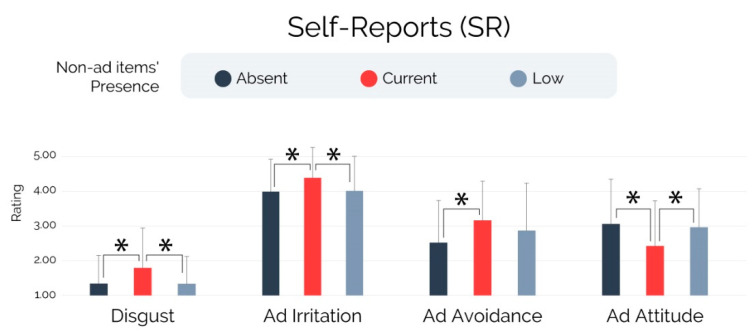
Self-reports. Note: Error bars represent standard deviation. Asterisks indicate statistically significant differences for *p* < 0.005.

The analyses performed on the self-reported measures of interest, illustrated in Figure 5 and based on the non-parametric ANCOVA, showed a significant effect of non-ad items’ presence on self-reported disgust (F = 4.762, *p* = 0.01, r^2^ = 0.056), ad irritation (F = 4.32, *p* = 0.015, r^2^ = 0.051), ad avoidance (F = 3.65, *p* = 0.028, r^2^ = 0.044), and ad attitude (F = 5.441, *p* = 0.005, r^2^ = 0.064). Regarding the self-reported disgust, subjects exposed to the “Current” condition exhibited significantly higher scores than those exposed to the “Absent” (p_scheffe_ = 0.026, r = 0.525) and “Low” (p_scheffe_ = 0.038, r = 0.492) conditions, while no statistical differences were found during the comparison “Absent”–“Low” (p_scheffe_ = 0.985). Concerning the self-reported ad irritation, subjects exposed to the “Current” condition exhibited significantly higher scores than those exposed to the “Absent” (p_scheffe_ = 0.03, r = 0.514) and “Low” (p_scheffe_ = 0.039, r = 0.45) conditions, while no statistical differences were found during the comparison “Absent”–“Low” (p_scheffe_ = 0.946). Focusing on the self-reported ad avoidance, subjects exposed to the “Current” condition exhibited significantly higher scores than those exposed to the “Absent” (p_scheffe_ = 0.029, r = 0.515), while no statistical differences were found during the comparisons “Current”–“Low” (p_scheffe_ = 0.554) and “Absent”–“Low” (p_scheffe_ = 0.285). Finally, the investigation conducted on the self-reported ad attitude showed that subjects exposed to the “Current” condition exhibited significantly lower scores than those exposed to the “Absent” (p_scheffe_ = 0.011, r = −0.585) and “Low” (p_scheffe_ = 0.038, r = −0.493) conditions, while no statistical differences were found during the comparison “Absent”–“Low” (p_scheffe_ = 0.893).

## 4. Discussion

### 4.1. Summary

YouTube, with its advertising campaigns potentially reaching 2 billion monthly users, is acknowledged as a key promotional tool by brands of any sector [3,4,5]. However, concerning its crown jewel, referred to as in-stream advertising, objective evaluations of viewers’ visual attention and emotional responses are lacking, leaving advertisers partially unaware of the effectiveness of their investments. Recently, some authors [123] have figured out, through self-reported measures, that the elements that YouTube overlays on the content of an in-stream ad, such as the countdown, can affect key advertising measures (e.g., ad irritation), while others [59], by applying the eye-tracking technique, were able to provide the first clear cues of how some of the YouTube non-ad items (e.g., skip button) can strongly grab the viewers’ attention, leading to a significantly decreased focus on the ad content. In addition, the same authors have shown how the non-ad items’ presence positively or negatively affects advertising measures depending on other specific ad features (e.g., ad position and length), highlighting, therefore, the need for caution when generalizing results and for additional studies aimed at extending current knowledge concerning in-stream advertising.

In order to overcome some of the existing limitations and extend current knowledge, the study’s authors, as detailed in this paper, employed remote neuromarketing techniques (webcam-based facial coding and eye-tracking) in combination with self-reports, to investigate the effect of the non-ad items’ presence on the viewers’ visual attention and disgust facial expressions, as well as on several key advertising measures, during the exposure to the YouTube 15-s, mid-roll, non-skippable in-stream ad format (the rationale underlying the choice to focus on this highly specific ad format is detailed in Section 1). The adopted between-subjects experimental design, which involved exposing participants to different levels of non-ad items’ presence (“Absent”, “Current”, “Low”) provided valuable insights, free of potential confounding effects (see covariates in Appendix A) and concerning all the measures of interest.

In particular, the eye-tracking analysis (RQ1) revealed that subjects exposed to the non-ad items currently employed by YouTube in overlay to the ad content (“Current” condition) as well as those exposed to an alternative version of the non-ad items whose presence has been experimentally reduced and customized (“Low” condition) paid significantly lower visual attention to the key ad elements (brand and product AOIs), and took significantly longer to notice such key ad elements than subjects not exposed to the non-ad items (“Absent” condition). No differences were found between the “Current” and “Low” groups regarding the total visual attention allocated to the key ad elements, despite the “Current” group paying significantly higher visual attention to the non-ad items (referred also to as “YouTube elements”) than the “Low” group. As a result, the presence of the non-ad items in their current or reduced forms contributes to decreasing the visual attention towards elements of the ad, such as brand or product items, whose notability is known to positively affect crucial advertising measures (e.g., ad and brand attitude and purchase intention) [72]. It is worth highlighting that subjects exposed to the non-ad items design currently employed by YouTube (“Current” condition) exhibited a decrease of 14.5% in total visual attention allocated to the key ad elements in comparison to those who viewed the ads without non-ad items presence (“Absent” group). The eye-tracking findings significantly contributed to decoding viewers’ gaze behavior in the context of YouTube non-skippable in-stream advertising, which lacks similar evidence, and where the effect of the non-ad items’ presence on eye-tracking measures concerning key ad elements was not previously explored.

The findings that emerged from the facial coding investigation revealed a significantly higher occurrence of facial expressions of disgust in subjects exposed to the “Current” condition in comparison to those exposed to the “Absent” and “Low” conditions. Furthermore, a significantly higher occurrence of facial expressions denoting disgust was found in the “Low” group in comparison to the “Absent” group. These results appear to provide little support for the hypothesis that a more pronounced presence of non-ad-elements would lead viewers to perceive the violation (e.g., forced ad view) less negatively, due to the higher transparency of the ad intent. In contrast, such findings seem highly consistent with the theories underlying memory, emotion, and neural interconnections in semantic networks, according to which the activation of an object’s representation is capable, regardless of the reasoning, of automatically eliciting the associated emotional responses [124,125,126,127]. Based on this framework, the non-ad items’ design currently adopted by YouTube that viewers are familiar with may serve as the primary distinguishing characteristic of an advertising format acknowledged for arousing the viewer’s feeling of having been deceived (e.g., “You can’t force me to watch those ads!” [116]), leading to a higher saliency of the violation and consequently to a more prominent occurrence of disgust-related facial expressions. In other words, the more the advertising format differs from the one currently used by YouTube, the less the participants manifest disgust, due to the lower presence of elements capable of activating the mental representation of the YouTube current ad format and the associated negative emotions, the latter due to the perceived deception and strengthened over time based on repeated exposures.

As well as the findings relying on eye-tracking and facial coding, which represent the primary focus of this paper, it is worth mentioning the main insights related to the self-reported measures of interest. In particular, subjects exposed to the ad format including the current YouTube non-ad items’ design (“Current” condition) reported significantly higher levels of disgust, ad irritation, and more unfavorable ad attitude than those exposed to the version including only the ad content without any overlaying non-ad items (“Absent” condition) and those exposed to the version in which the non-ad items presence resulted reduced (“Low” condition). Moreover, the measured levels of self-reported ad avoidance evidenced that the “Current” group adopted advertising avoidance strategies (ad avoidance) to a significantly greater extent than the “Absent” group, while no differences in this direction were found between the “Current” and “Low” groups as was the case with the other self-reported measures.

Considering the overall findings, the non-skippable ad format currently employed by YouTube in mid-roll position (“Current” condition) performed worse than its equivalent without elements superimposed on the advertising content (“Absent” condition) on all the investigated measures and than its equivalent featured by a reduced non-ad items’ presence (“Low” condition) on facial coding disgust, self-reported disgust, ad irritation, and ad attitude. Beyond the saliency of the deception which may have played a primary role in our context according to the theories underlying memory, emotion, and neural interconnections in the semantic network [124,125,126,127], further effects due to the specific nature of some of the non-ad items may have arisen. In this direction, it is crucial to highlight that the element providing the time certainty in the “Current” condition was a countdown timer, while in the “Low” condition was a static text. As elements reporting temporal information are particularly salient in non-skippable ads and the countdown timer, which was available only for the “Current” group, has been associated with “time anxiousness” by previous research [151,152], it may be plausible to take into account such additional aspects while interpreting the differences between the “Current” and “Low” group. Differences between in-stream ads including the countdown timer or a text providing static temporal information were explored in detail by the study of Jeon et al. (2019) [123], whose results did not support their prediction that the countdown timer elicited higher ad irritation than the static text. However, as their study focused on pre-roll ads, which are known to be perceived differently than mid-roll ads (the objects of our investigation) [59], future studies may provide further clarification in this direction.

The present study made significant contributions to the existing body of knowledge in the field of in-stream advertising across several dimensions. First, the understanding of how viewers direct their attention to key ad elements (e.g., brand and product AOIs) according to different levels of non-ad items’ presence was provided in the context of YouTube in-stream advertising, where only one study conducted similar investigations so far, however focusing on the general content of the ad [59]. Second, despite emotions being known to play a crucial role in advertising [79,80,81,82], emotional responses in the context of YouTube in-stream advertising have been little explored, and in particular, no contributions concerning the role of disgust, which is a primary emotional mediator of advertising effects [88,89,90,91], and/or based on the facial expression analysis (FEA) were provided to shed light on the issue addressed by this paper. In this context, the application of facial coding for the investigation of moral disgust during the view of in-stream ads, due to its sensitivity to nonbodily violations (e.g., betrayal or deception) and moralistic anger [102,103], contributed to the emergence of valuable findings, whose interpretations are consistent with the proposed framework [124,125,126,127]. In addition, unlike other measures, disgust-related facial expressions proved to be the most responsive cues to the variation of the non-ad items’ presence, as significant differences emerged on all multiple pairwise comparison tests.

Third, this study provided further evidence of how the integration of traditional and neuromarketing techniques can enable researchers to obtain a more in-depth knowledge of the consumer. In this direction, it is also worth confirming the advantages concerning remote data collection relying on webcam-based eye-tracking and facial coding, whose standards of precision are becoming more stringent, and that can be enabled through the simple use of participants’ digital devices, preserving ecological validity and potentially involving participants from all over the world [36,37,38].

Finally, it is essential to highlight the most relevant implications for both professionals and researchers operating in advertising. Although pre-testing is undoubtedly a fundamental practice for assessing the effectiveness of ads in advance and decreasing the likelihood of post-launch failures, our findings strongly demonstrated that external or contextual factors, if not taken into account, may drastically affect the expected outcomes.

For instance, if an advertisement has been designed to serve a YouTube 15-s, mid-roll, non-skippable in-stream ad format, it is critical to pre-test its effectiveness in a format that resembles the target one and that includes the current YouTube non-ad items’ design. That is because our findings revealed that the ad, when presented without non-ad items’ presence (“Absent” condition), performed significantly better on all the investigated measures than that one including the non-ad items’ presence as currently adopted by YouTube (“Current” condition). The question that arises spontaneously is: how often are these factors taken into consideration during a pre-test? If taking these factors into consideration may result in increased costs, longer time, and more effortful development, it may also contribute to achieving more accurate estimates. After all, manipulating variables that one might think have little effect has been shown to produce dramatically different results (e.g., same ad format while varying its occurrence in pre-roll or mid-roll position) in YouTube in-stream advertising [59]. Accordingly, this is the main reason that led us to narrow down the investigation to a highly specific format, suggesting a reasonable level of confidence in the generalizability of our findings to real-world contexts, specifically in relation to the YouTube 15-s, mid-roll, non-skippable in-stream ad format.

Moreover, YouTube, as well as other providers operating in in-stream advertising, may consider that the current design of the elements aiming at disclosing the advertising intent and overlaying on the ad content may lead to more unfavorable outcomes (e.g., facial coding and self-reported disgust, ad irritation, and ad attitude) in comparison to alternative designs where such features are reduced and/or rearranged, presumably due to the higher saliency of the deception associated with the current and familiar format. Beyond the theories underlying memory, emotion, and neural interconnections in the semantic network [124,125,126,127], and those highlighting the potential effect of specific non-ad items (e.g., countdown timer inducing “time anxiousness”) [151,152], which support the proposed interpretations, the increasing abilities of online users in identifying ads through distinct features as well as their persuasive intent should not be overlooked, since it represents a major issue for the advertising industry, known to significantly weaken advertising performance [44,45,46,47,48,51].

### 4.2. Recommendations for Future Experimental Studies

Some limitations of the study and recommendations for future research are discussed below. Whereas the specificity of our context was essential to address our research questions, it is worth emphasizing once again that the generalization of our findings is limited to the YouTube 15-s, mid-roll, non-skippable in-stream ad format. Additional studies might therefore investigate the effect of the non-ad items’ presence in further scenarios, varying the platform, ad position (e.g., pre-roll), the ad content, skipping characteristic (skippable/non-skippable), ad length (according to the country where the study is conducted [25,73]), and other features. Furthermore, regarding the remote collection of eye-tracking and facial coding data, to obtain a valid sample of 175 participants, we had to record a significantly higher number of subjects. This issue was due to several factors preventing an accurate measurement such as the poor webcam hardware, a dated operating system, and bad lighting conditions (e.g., poorly lit face or strong light source behind). According to our estimates, to get a valid sample of 50 participants for an experimental design similar to that described in this paper, it may be necessary to record approximately 100 participants via desktop, or 200 participants via smartphone. While the remote collection of eye-tracking and facial coding data may be still more cost-effective than its “in-lab” counterparts, these issues prevented us from obtaining the valid sample we were aiming for within the timeframe available, i.e., a valid sample of 300 individuals, where within each condition we aimed to include 50 desktop users and 50 smartphone users, which would have allowed us to carry out in-depth analysis at the device level. Moreover, it prevented us from achieving an identical sample size across conditions or within conditions at the device level. In summary, it is worth noting that the remote data collection performed with webcam-based techniques may not proceed as planned, in particular when the test is administered by smartphone, and due to the intervention of external factors that are beyond the researcher’s control and that will inevitably need to be addressed through future technological advancements.

Another limitation relies on the ad content employed. Unfortunately, it was obviously not possible to provide users with a relevant ad according to YouTube’s algorithms. However, as YouTube reported that “the ads you see may be based on the content of the video you’re viewing” [153], we decided to employ a video and an advertisement that shared key similarities. In particular, both were focused on the subject of food and more specifically on chocolate-covered food. More complex strategies, such as the development of a website resembling YouTube and allowing the selection of videos based on participants’ interests could not be adopted, due to the incompatibility between such highly interactive environments and the tools used for remote test administration.

At the same time, it is crucial to mention that people might not feel as disgusted in an experiment as they would in real life due to the forced exposure to the video, which was one of the experimental features. In particular, subjects had to watch a video on “how to make a mug cake in the microwave” whether they found it interesting or not and this may have produced changes in their initial reaction of disgust when the ad showed up, just like it may occur in real life. Future research on this topic may therefore consider adopting an ad-hoc platform resembling the original one where participants are allowed to select one or more videos of interest to watch, reflecting higher control over their choices and even more confidence concerning the generalizability of the results to the real world. In a similar context, participants will be able to pick a video that interests them, and the emotional response associated with the in-stream ad may be more genuine.

While such approaches would better fit the principles underlying YouTube ad targeting and can be currently employed in “in-lab” studies, new technological advances are needed to enable the application of similar procedures in combination with eye-tracking and facial coding remote technologies. Progress in this direction would provide new and exciting perspectives for the future of neuromarketing and consumer neuroscience.

## 5. Conclusions

The primary objective of the present study was to investigate the effect of the elements that YouTube overlays on the content of the advertisements on viewers’ attention towards key ad elements (e.g., brand and product) as well as on their facial expressions of disgust which have been shown to be highly responsive to the abstract violations of moral norms as it is the forced ad exposure following the unexpected interruption that characterizes the video viewing experience on YouTube. The results obtained from these measures and the self-reported measures traditionally investigated in advertising research evidenced a clear effect of such YouTube elements overlaid on advertising content.

In a context featuring rapid expansion and valuable advertising opportunities, the findings provided by this paper contribute to increasing the understanding of the dynamics that affect the consumer at implicit and behavioral levels during the view of in-stream advertising on YouTube. In addition, several implications for both professionals and researchers operating in advertising were discussed, as well as the benefits and drawbacks related to the application of remote neuromarketing techniques in addressing current and future challenges.

## Figures and Tables

**Figure A1:**
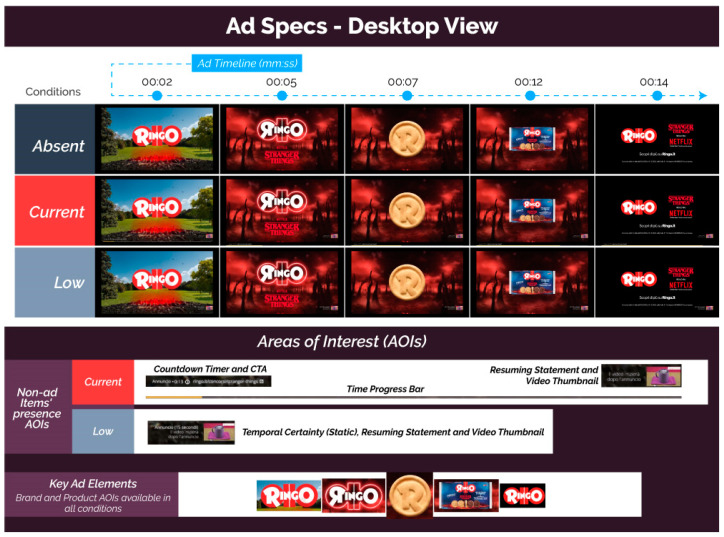
Ad specs—desktop view.

**Figure A2:**
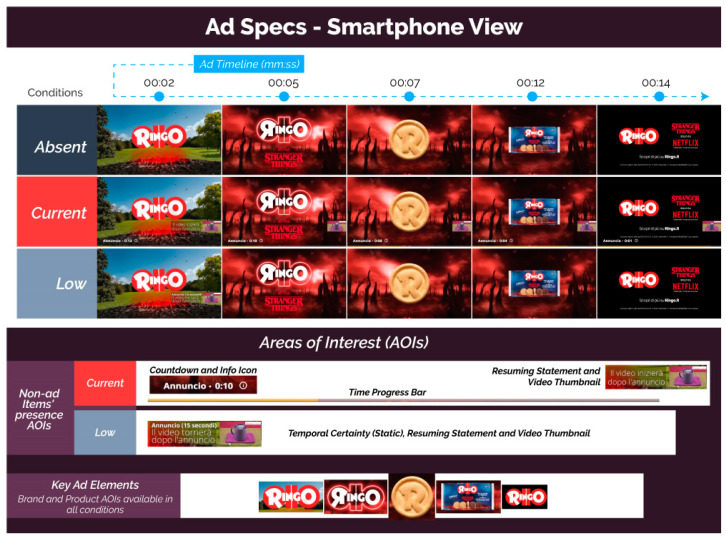
Ad specs—smartphone view.

## Data Availability

The data presented in this study are available on request from Prof. Babiloni (fabio.babiloni@brainsigns.com).

## Appendix A

### Appendix A.1. Sample Video Links

This section provides several video links of interest. To view the videos in the same mode as the experimental subjects, it is recommended to download the videos locally and view them in a video editing software in full-screen mode. This will help prevent various confounding elements introduced by YouTube from leading to biased views (duplicated video controllers, channel profile image at the bottom right).

References adopted for the ads’ development:

- Desktop (the ad starts at 00:02): https://www.youtube.com/watch?v=Y2ncWsOfcoE (accessed on 7 September 2022)
- Smartphone (the ad starts at 00:02): https://www.youtube.com/watch?v=SZu1EkFDWDM (accessed on 7 September 2022)

Example of the overall YouTube viewing experience including the video and the mid-roll ad (“Current” condition):

- Desktop: https://www.youtube.com/watch?v=il4JY2HmUkk (accessed on 7 September 2022)
- Smartphone: https://www.youtube.com/watch?v=h4SBJ8TGCY0 (accessed on 7 September 2022)

### Appendix A.3. Self-Reports

This section provides details about the self-reported measures of interest (disgust, ad irritation, ad avoidance, ad attitude) as well as other measures needed to control confounding in the analysis (YouTube video viewing frequency, food ads attitude, product attitude, product purchase frequency).

*Legend: Self-Reported Measure|Description|Rating|Role in the Analysis*

- **Disgust**|While watching the ad, I felt disgust|Strongly Disagree (1), Disagree (2), Neither agree nor disagree (3), Agree (4), Strongly Agree (5)|Dependent Variable
- **Ad Irritation**|The ad irritated me|Strongly Disagree (1), Disagree (2), Neither agree nor disagree (3), Agree (4), Strongly Agree (5)|Dependent Variable
- **Ad Avoidance**|While watching the ad I avoided looking at the ad content (e.g., focusing my attention on areas of the screen where they weren’t present)|Strongly Disagree (1), Disagree (2), Neither agree nor disagree (3), Agree (4), Strongly Agree (5)|Dependent Variable
- **Ad Attitude**|I liked the ad|Strongly Disagree (1), Disagree (2), Neither agree nor disagree (3), Agree (4), Strongly Agree (5)|Dependent Variable
- **YouTube video viewing frequency**|On average, how much time do you spend watching YouTube videos each week?|I don’t watch YouTube videos (1), Maximum 10 min (2), From 10 to 30 min (3), From 30 min to 1 h (4), 1 to 2 h (5), More than 2 h (6)|Covariate
- **Food ads attitude**|I like food ads|Strongly Disagree (1), Disagree (2), Neither agree nor disagree (3), Agree (4), Strongly Agree (5)|Covariate
- **Product attitude**|In general, I have a positive attitude toward the Ringo product|Strongly Disagree (1), Disagree (2), Neither agree nor disagree (3), Agree (4), Strongly Agree (5)|Covariate
- **Product purchase frequency**|How often do you buy the Ringo?|Never (1), Less than once a year (2), At least once a year (3), At least once every 6 months (4), At least once a month (5), At least once a week (6)|Covariate
